# Integrated metabolomics and gut microbiome to the effects and mechanisms of naoxintong capsule on type 2 diabetes in rats

**DOI:** 10.1038/s41598-020-67362-2

**Published:** 2020-07-02

**Authors:** Zenghao Yan, Hao Wu, Haokui Zhou, Shuo Chen, Yan He, Weijian Zhang, Taobin Chen, Hongliang Yao, Weiwei Su

**Affiliations:** 1 Guangdong Engineering and Technology Research Center for Quality and Efficacy Reevaluation of Post-Market Traditional Chinese Medicine, Guangdong Key Laboratory of Plant Resources, State Key Laboratory of Biocontrol, School of Life Sciences, Sun Yat-sen University, Guangzhou, 510275 People’s Republic of China; 2 Institute of Synthetic Biology, Shenzhen Institutes of Advanced Technology, Chinese Academy of Sciences, Shenzhen, 518055 People’s Republic of China; 3 Guangdong Key Laboratory of Animal Conservation and Resource Utilization, Guangdong Public Laboratory of Wild Animal Conservation and Utilization, Drug Synthesis and Evaluation Center, Guangdong Institute of Applied Biological Resources, Guangdong, 510260 People’s Republic of China

**Keywords:** Pharmacology, Drug development

## Abstract

Naoxintong Capsule (NXT) is a Traditional Chinese Medicine formulation which has been widely applied in treating cardiovascular and cerebrovascular diseases. Previous studies also reported the potential effects of NXT against diabetes and certain complications, yet its mechanisms remain largely obscured. Herein, in this study, we investigated the anti-diabetic effects of NXT as well as its potential mechanisms. Type 2 diabetes (T2D) was induced in rats by 10-week high-fat diet in companion with a low-dose streptozotocin injection. NXT was administrated for additional 8 weeks. The results showed that NXT exerted potent efficacy against T2D by alleviating hyperglycemia and hyperlipidemia, ameliorating insulin resistance, mitigating inflammation, relieving hypertension, and reducing myocardial injuries. To investigate its mechanisms, by integrating sequencing of gut microbiota and serum untargeted metabolomics, we showed that NXT could significantly recover the disturbances of gut microbiota and metabolic phenotypes in T2D rats. Several feature pathways, such as arachidonic acid metabolism, fatty acid β-oxidation and glycerophospholipid metabolism, were identified as the potential mechanisms of NXT in *vivo*. In summary, our study has comprehensively revealed the anti-diabetic effects of NXT which could be considered as a promising strategy for treating metabolic disorders, T2D and diabetic related complications in clinical practice.

## Introduction

Type 2 diabetes (T2D), pathologically characterized by insulin resistance, pancreatic β-cell dysfunction and low-grade inflammation, is a serious public health issue and threat globally^1^. The prevalence of diabetes in adults was estimated to be 8.4% in 2017 and predicted to rise to 9.9% in 2045^2^, among which T2D accounts for over 90% of the population^3^. T2D leads to a series of complications, such as atherosclerosis, hypertension, ischemic stroke, diabetic nephropathy et al*.*, which together contribute to high incidences of morbidity and mortality^4^. Hence, therapeutic strategies for T2D and its complications are of great significance and demand in clinical practice. Anti-diabetic drugs, including insulin, insulin sensitizers, insulin secretagogues and glucose absorption inhibitors^5^, have been widely used in treating hyperglycemia and related complications, yet many of which have various side effects such as chronic nephrotoxicity and gastrointestinal discomforts. Traditional Chinese Medicine (TCM), in contrast, had been applied as an alternative strategy to treat metabolic disorders through thousand years of practice with its unique advantages of good efficacy and safety^6^.

Naoxintong Capsule (NXT) is a well-known TCM formulation modified from classic formula Buyang Huanwu Decoction of Qing Dynasty. It is a fine powder mixture of 11 medicinal herbs (*Astragali Radix* (Huangqi), *Paeoniae Radix Rubra* (Chishao), *Salviae miltiorrhizae Radix et Rhizoma* (Danshen), *Angelicae sinensis Radix* (Danggui), *Chuanxiong Rhizoma* (Chuanxiong), *Persicae Semen* (Taoren), *Achyranthis bidentatae Radix* (Niuxi), *Spatholobi Stem* (Jixueteng), *Cinnamomi Ranulus* (Guizhi), *Carthami Flos* (Honghua), and *Mori Ramulus* (Sangzhi)), 2 resin medicines (*Olibanum* (Ruxiang), *Myrrha* (Moyao)), and 3 animal medicines (*Scorpio* (Quanxie), *Pheretima* (Dilong), and *Hirudo* (Shuizhi))^7^. In China, NXT has been approved by China Food and Drug Administration (CFDA) to treat cardiovascular and cerebrovascular diseases and showed significant therapeutic effects and high safety in over two decades of clinical application^8–11^. In recent years, NXT has been applied in T2D patients to improve vascular functions and ameliorate hyperglycemia^12^. Previous studies have also shown that NXT could improve glucose metabolic disorders and rescue renal functions in *db/db* mice^13,14^. Although several studies have mentioned the anti-diabetic effects of NXT, its mechanisms and actions on T2D remain largely obscured.

The development of T2D and its complications is associated with a complex pathogenesis, including dynamic changes in host metabolism^15^. In recent years, emerging evidence have suggested that gut microbiota plays a vital role in host immunity and metabolism with imbalances of gut microbiota observed from patients with metabolic and cardiovascular diseases^16,17^. These effects have partly been proven to be mediated through metabolites. Gut microbiota may be the origin of the abnormal serum metabolites previously associated with T2D and its complications^18,19^. More optimized therapeutic strategies, especially TCM, have been taken to target the gut microbiota to combat diseases and improve health with a deeper understanding of these relationships^20,21^. NXT is composed of 16 TCM herbs in total. Up to 178 components were identified or tentatively characterized in NXT, including flavonoids, phenanthraquinones and terpenoids^22^. Our group also established a chemical profile of NXT in vivo using bloods and feces of beagle dogs^23^. It is reported that some metabolites could be regulated by the direct effects of NXT’s compositions^24–27^. In addition, there are a large number of components hardly absorbed like dietary fibers in NXT and these components are likely to affect gut microbiota^23^. Therefore, in order to characterize the global effects and mechanisms of NXT to treat T2D and its complications, it is interesting to combine research into gut microbiota with metabolomics.

In this study, by integrating serum untargeted metabolomics and sequencing of gut microbiota, we aimed to investigate the beneficial effects of NXT on T2D and its complications as well as its potential mechanisms. We reported the protective effects of NXT to T2D via alleviating hyperglycemia and hyperlipidemia, ameliorating insulin resistance, mitigating inflammation, relieving hypertension as well as reducing myocardial injuries. To study the mechanisms, we revealed that several feature metabolic pathways and gut microbiota were highly associated with the beneficial outcomes of NXT. Our study emphasized the potent efficacy of NXT which could be considered as a promising strategy to treat T2D and diabetic complications in clinical practice.

## Material and methods

### Materials and reagents

Naoxintong Capsule (NXT) (lot No. 170589) was provided by Shanxi Buchang Pharmaceutical Co., Ltd. (Xianyang, China). Myristic acid-D27 (98%) was purchased from Cambridge Isotope Laboratories, Inc. (Andover, USA). MS grade formic acid, streptozotocin (STZ) and glucose were purchased from Sigma-Aldrich Co. (St. Louis, USA). Insulin was obtained from Novo (China) Pharmaceutical Co., Ltd. (Beijing, China). Rodent standardized diet was provided by Guangdong Medical Laboratory Animal Center (Foshan, China). Rodent high-fat diet (HFD) with 60 kcal% fat was purchased from Research Diets Inc. (D12492, New Brunswick, USA). MS grade methanol and acetonitrile were purchased from Thermo Fisher Scientific Inc. (Fair Lawn, USA). Deionized water was purified by the Milli-Q system (Millipore, Merck KGaA, Darmstadt, Germany) and filtered through 0.22 mm membrane filter prior to use.

### Animals and treatment

Animal protocols in this study were supervised and approved by the Ethics Committee of School of Life Sciences, Sun Yat-sen University. All animal experiments were carried out in strictly accordance with the guidelines and regulations for the care and use of laboratory animals of School of Life Sciences, Sun Yat-sen University. Every effort was made to minimize both the employed animal numbers and the potential discomforts of the animals throughout the study.

Male Sprague–Dawley rats (specific pathogen free grade) with body weights from 180 to 220 g were purchased from Guangdong Medical Laboratory Animal Center (Certificate No. 44007200040426, Foshan, China). Animals were housed in a standardized pathogen free area with ambient temperature (23 ± 3 ℃)/humidity (55 ± 15%) control and a 12/12 h light/dark cycle.

A traditional T2D model was induced by HFD feeding in combination with a low-does STZ injection as described previously with minor modifications^28–30^. Briefly, rats were divided into sham group (n = 10) and HFD group (n = 20) with standardized normal diet and HFD feeding for continuous 10 weeks, respectively. On day 54, sham and HFD rats received one dose intraperitoneal injection of citrate buffer (ice cold, pH4.5) and STZ (30 mg/kg in ice cold citrate buffer, pH4.5), respectively. 72 h post injection, fast blood glucose (FBG) levels were measured and those rats with FBG levels no less than 11.0 mM were recruited for further experiments.

To evaluate the effects of NXT, we randomly divided the recruited diabetic rats into 2 groups (10 per group). The treatments were administrated daily for additional 8 weeks based on the clinical usage as follows: (1) NXT group: recruited T2D rats were fed with HFD and treated with NXT (1,000 mg/kg/day) by intragastric administration; (2) model (MOD) group: recruited T2D rats were fed with HFD and administrated with the same volume of normal saline; (3) control (CON) group: sham rats were fed with standardized normal diet and administrated with the same volume of normal saline. Throughout the study, rats were fed ad libitum and we routinely monitored bodyweight, food intake, water drinking and exterior appearance. At the end of the experiment (day 127), all the rats were sacrificed after overnight fasting followed by collection of blood and serum samples. The blood samples were immediately used for biochemical analysis and serum samples were stored at − 80 °C until the following analysis.

### Oral glucose tolerance test (OGTT) and insulin tolerance test (ITT)

Rats underwent an OGTT and an ITT in week 18 by methods described in our previous article^29^. For OGTT, after fasting for 12 h, rats were challenged with 3.0 g/kg glucose intragastrically. Blood samples were collected at 0, 15, 30, 45, 60, 90, 120 min post administration via tail vein. For ITT, after fasting for 12 h, 0.75 IU/kg of insulin was injected intraperitoneally. Blood samples were collected at 0, 15, 30, 45, 60, 90, 120 min post injection via tail vein. The blood glucose levels were measured using OneTouch® UltraVue glucometer (Johnson & Johnson Services, Inc., New Brunswick, USA).

### Measurement of blood pressure

The blood pressures of rats were measured on day 126 by indirect tail-cuff method using BP-2010A system (Softron Inc., Tokyo, Japan) according to the manufacturer’s instruction. The systolic blood pressure (SYS), diastolic blood pressure (DIA) and mean arterial pressure (MAP) of each rat were measured in triplicate.

### Biochemical analysis

The glycated hemoglobin A1c (HbA1c) levels were measured in whole blood samples using HbA1c assay kit from Shanghai Kehua Bio-engineering Co., Ltd. (Shanghai, China) and Hitachi 7020 automatic biochemical analyzer (Tokyo, Japan). Serum levels of glucose (GLU), free fatty acids (FFAs), high-density lipoprotein cholesterol (HDL-C), low-density lipoprotein cholesterol (LDL-C), total cholesterol (TC), total glyceride (TG), hydroxybutyrate dehydrogenase (α-HBDH), creatine kinase (CK), creatine kinase isoenzyme (CK-MB) and lactate dehydrogenase (LDH) in rat serum samples were measured and quantified using colorimetric kits (Nanjing Jiancheng Bio-engineering Institute, Nanjing, China) according to manufacturer’s instructions. Serum levels of interleukin-1β (IL-1β), interleukin-4 (IL-4), interleukin-6 (IL-6), tumor necrosis factor-α (TNF-α), insulin (INS), angiotensin-2 (ANG-2), C reactive protein (CRP) and endothelin 1 (ET-1) were measured using ELISA kits (Cloud-Clone Corp., Katy, USA) according to manufacturer’s instructions.

### Fecal DNA isolation

In this study, DNA isolation and V4-V5 16s rRNA gene region amplification and sequencing of fecal samples followed those protocols described previously by our group^29^. On day 126, 1 g fecal samples of each rat were collected. Fecal DNA was extracted using PowerSoil DNA isolation kit (MO BIO Laboratories, QIAGEN, Dusseldorf, Germany) according to manufacturer’s instructions. The integrity of extracted DNA was verified by 1% agarose gel electrophoresis.

### V4-V5 16s rRNA gene region amplification and sequencing

The hypervariable V4-V5 regions of the 16S rRNA genes from bacteria and archaea were amplified in triplicate by PCR using the barcoded primers 515-F (5′-GTGCCAGCMGCCGCGGTAA-3′) and 907-R (5′-CCGTCAATTCMTTTRAGTTT-3′) (Invitrogen, Thermo Fisher Scientific Inc., Fair Lawn, USA). PCR was performed on a thermocycler using Premix Taq (EX Taq Version 2.0 plus dye, Takara Biotechnology Co. Ltd., Dalian, China) with following conditions: 94 °C for 5 min followed by 30 cycles of 94 °C for 30 s, 52 °C for 30 s, and 72 °C for 30 s and a final extension at 72 °C for 10mins. The PCR products were verified by 1% agarose gel electrophoresis and quantified using GeneTools analysis software (Version 4.03.05.0, SynGene, Frederick, USA). Technical triplicate amplicons were pooled and gel purified using EZNA Gel Extraction Kit (Omega Bio-tek, Inc., Norcoross, USA). Sequencing was performed using 250-bp paired-end strategy on an Illumina HiSeq 2500 platform (Illumina Inc., San Diego, USA) with NEBNext® Ultra™ DNA Library Prep Kit for Illumina® (New England Biolabs, Ipswich, USA).

### Sequencing data analysis

Sequencing data analysis was performed with QIIME 2 2020.2 (https://qiime2.org)^31^ and R 3.6.3 (https://www.r-project.org), and we used a modification of the method described in Pearson et al. ^32^. Raw pair-end sequencing data were demultiplexed using q2-demux plugin and the demultiplexed sequences were merged and quality filtered using q2-cutadapt, q2-vsearch and q2-quality filter plugins. After denoising with Deblur^33^ (via q2-deblur), all amplicon sequence variants (ASVs) were aligned with mafft^34^ (via q2-alignment) and used to construct a phylogeny with fasttree2^35^ (via q2-phylogeny). The rarefaction curve, α-diversity metrics (Pielou’s evenness index, observed OTUs, Faith’s phylogenetic diversity and Shannon index), β-diversity (unweighted unifrac and Bray–Curtis dissimilarity), and principle coordinate analysis (PcoA) were estimated using q2-diversity plugin. ASVs were annotated with taxonomic information using the Naïve Bayes classifiers trained on Greengenes 13_8 99% OTUs sequences (via q2-feature-classifier)^36,37^. Adonis analysis based on distance matrix was performed using vegan package (https://CRAN.R-project.org/package=vegan). A random forest classification model was trained on the taxonomy data using Boruta package to classify features (https://CRAN.R-project.org/package=Boruta)^38^. Significant difference of the selected features among tested groups was then determined using the Mann–Whitney U test (wilcox.test()). Spearman’s correlation analysis was performed to identify the correlations using psych package (https://CRAN.R-project.org/package=psych). The P values were corrected to P.adj values by FDR method (Benjaminiand-Hochberg method) (p.adjust()).

### Untargeted metabolomics analysis

To prepare samples for untargeted metabolomic analysis, 100 μL serum was thawed and mixed with 400 μL ice cold methanol/acetonitrile (1:1, *v/v*) containing 20 μg/mL of myristic acid-D27 as internal standard (IS). The mixture was then vortexed for 30 s and incubated at -20 °C for 1 h for complete protein precipitation. After centrifugation at 15,000*g* for 20 mins, 200 μL supernatant was collected and 10 μL of the supernatant was injected for analysis. Quality control (QC) samples were prepared by blending equal volume (5 μL) of each sample. And pooled QC samples were injected every eight samples during detection in order to monitor and adjust system stability.

Untargeted metabolomics analysis was conducted on a connected system of UFLC XR (Shimadzu Corp., Kyoto, Japan)-hybrid triple quadruple time-of-flight mass spectrometer (Triple TOF™ 5600+ , AB Sciex, Forster City, USA) with electrospray ionization source. Chromatographic separation was performed on 1.8 μm 2.1 × 100 mm ACQUITY UPLC® HSS T3 column (Waters, Milford, USA) at 50 °C with a flow rate of 0.3 mL/min. The elution gradient program with solvent A (deionized water with 0.1% formic acid) and solvent B (acetonitrile with 0.1% formic acid) was set as follows: 0–1.5 min, isocratic 1% B; 1.5–13 min linear gradient from 1 to 99% B; 13–16.5 min isocratic 99% B, and afterward back to 1% B in 3.5 min. The instrumental settings of Q-TOF–MS/MS were set as recommended by AB Sciex: ion source gas 1 and gas 2 were both 55 psi, curtain gas was 35 psi, ion source temperature was 550 °C, ion spray voltage floating was 5,500 V in positive mode while − 4,500 V in negative mode, collision energy was 30 V, collision energy spread was 15 eV, and declustering potential was 80 V. Nitrogen was used as nebulizer and auxiliary gas. Serum samples were analyzed in both positive and negative ionization modes with scanning mas-to-charge (m/z) range from 50 to 1,500 Da. Data were collected using Analyst® software (AB Sciex, Foster City, USA) in information-dependent acquisition (IDA) mode.

To analyze metabolomic data, the mass spectrum data were processed by PeakView® 2.2 and Markerview 1.2 software (AB Sciex, Foster City, USA) for noise reduction and peak alignment and identification. Peak intensities were normalized by IS. To identify differential metabolites, orthogonal partial least square-discriminant (OPLS-DA) analysis was performed by SIMCA-P 14.1 (Umetrics AB, Umea, Sweden), and feature metabolites among groups was characterized by variable importance value (VIP) > 1 and a value of *P* < 0.05 (significance test). These feature metabolites were further identified or tentatively characterized by mass fragmentation patterns in comparison with databases including Chemspider (www.chemspider.com) and HMDB (www.hmdb.ca)^39^. The metabolic pathway identification and analysis were applied by MetaboAnalyst 4.0 (www.metaboanalyst.ca)^40^.

### Statistical analysis

Statistical analysis was performed as described with minor modification^29^ using SPSS statistical software (Version 22.0, SPSS. Inc, Chicago, USA), and GraphPad Prism 8 (San Diego, USA). The data were presented as the mean ± SD. Significance tests among groups was performed using one-way ANOVA followed by Tukey’s post hoc test only when the data were normal and variances were equal. Otherwise, the Mann–Whitney U test was used. A P value of < 0.05 was considered significant statistically.

## Results

### NXT alleviated crucial diabetic phenotypes in T2D rats

**Figure 1:**
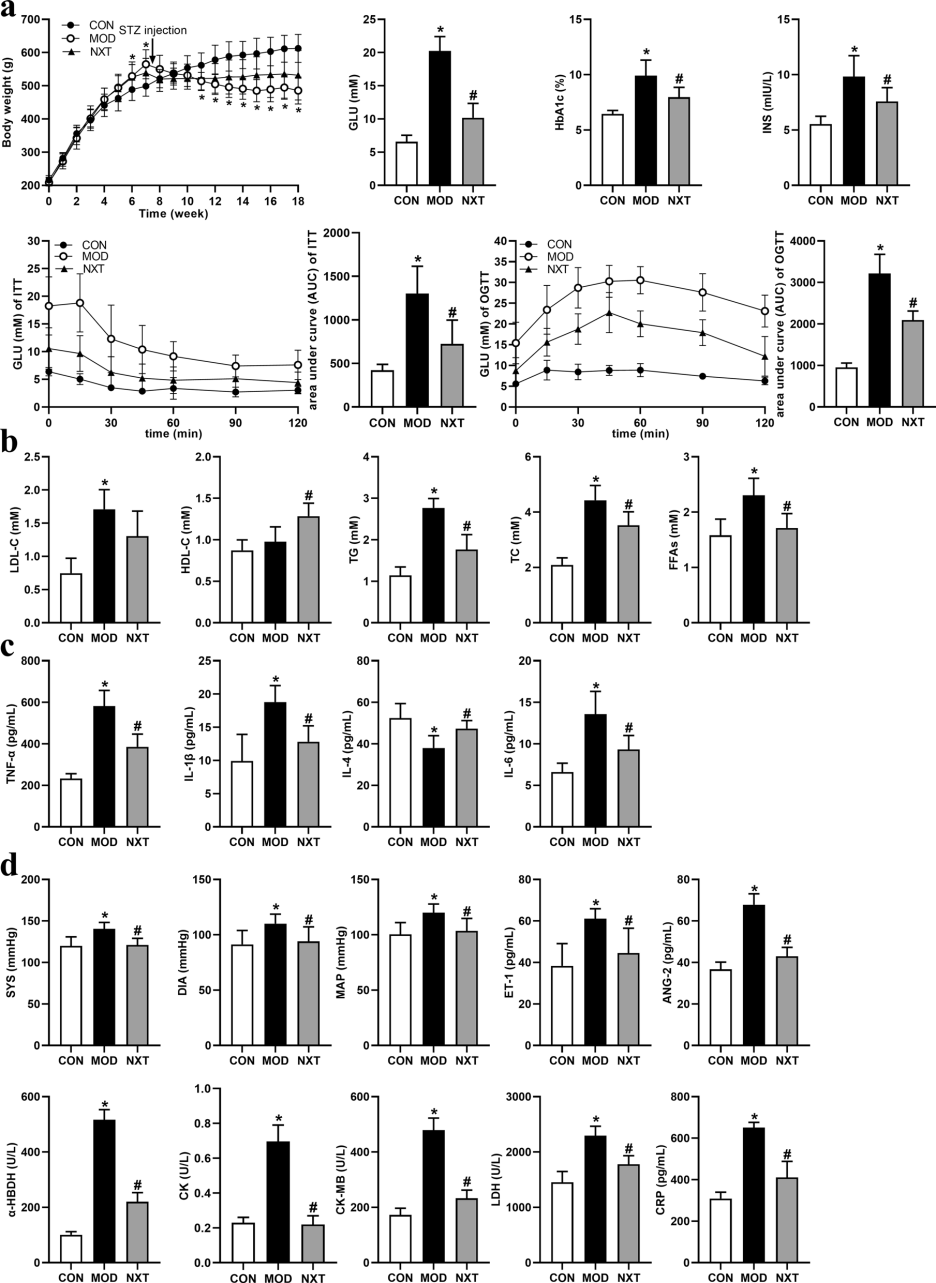
The effects of NXT on body weight and glucose metabolism (**a**), lipid metabolism (**b**), inflammatory response (**c**) and cardiovascular system (**d**) in T2D rats. Data were presented as mean ± SD (n = 10 in each group). CON, control group; MOD, model group; NXT, NXT-treated group. *P < 0.05 vs. CON group; ^#^*P* < 0.05 vs. MOD group by Tukey’s post hoc test.

We first evaluated the anti-diabetic effects of NXT. A T2D model in rats was induced by 10-week HFD. On day 54, rats received a single dose injection of STZ (30 mg/kg) and those rats with FBG levels no less than 11.0 mM were recruited for further experiments. Diabetic rats then received NXT (1,000 mg/kg) or vehicle administration for continuous 8 weeks and blood samples were collected for evaluation of diabetic phenotypes at the end of the treatment. As shown in Fig. 1a, rats fed with HFD dramatically gained body weights in the first six weeks when compared to control rats fed with chow diet (*P* < 0.05). After STZ injection on day 54, however, HFD rats showed a continuous decline of body weights (*P* < 0.01), which partially resulted from the injuries of insulin producing β-cells in pancreatic islets and metabolic disturbance. Notably, NXT could recover the abnormal changes of body weights of T2D rats. We then conducted OGTT and ITT among three groups. 8-week NXT treatment was capable of improving glucose metabolism not only by enhancing insulin resistance, but by lowering fasting serum GLU, fasting INS and HbA1c levels as well (*P* < 0.05) (Fig. 1a). As for lipid metabolism, T2D rats exhibited hyperlipidemia with marked increase levels of serum TC, TG, LDL-C and FFAs (*P* < 0.01), while NXT treatment effectively ameliorated the up-regulation of TG, TC, FFAs, LDL-C levels and increased HDL-C level (*P* < 0.05) (Fig. 1b). As for inflammatory response, serum TNF-α, IL-1β and IL-6 levels were significantly up-regulated while the serum IL-4 level markedly decreased in the MOD rats compared with the CON rats (*P* < 0.01). However, IL-1β, TNF-α and IL-6 levels were decreased and IL-4 level were increased in NXT-treated rats compared with the MOD rats (*P* < 0.05), which showed NXT could alleviate chronic inflammation with the development of T2D (Fig. 1c).

Diabetes damages blood vessels and majorly arteries, and causes pathological injuries such as atherosclerosis, hypertension and vascular dysfunctions. Of note, diabetic hypertension is a common symptom which can lead to severe diabetic complications including diabetic nephropathy, retinopathy, and even heart attack. To address this concern, we also evaluated the potential effects of NXT on diabetic hypertension and vascular functions. Rats that received long-term HFD feeding and single STZ injection showed hypertension phenotypes with increased SYS, DIA and MAP (*P* < 0.05). NXT exerted a potent hypertension lowering effect as reduced SYS, DIA and MAP levels, when compared to MOD group (*P* < 0.05) (Fig. 1d). ET-1 is a potent vasoconstrictor which actively involved in the pathogenic processes of hypertension, vascular remodeling, endothelial dysfunction and inflammation. Increasing evidences have highlighted the therapeutic significance of ET receptor antagonists in treating hypertension and related complications. In our study, we found that NXT may partially inhibited hypertension induced overexpression of ET-1, which may suggest its potential effect against vascular injuries (*P* < 0.01) (Fig. 1d). ANG-2 is another crucial participant in modulating vascular functions and inflammation. On one hand, ANG-2 could up-regulate ROS production, which in turn inactivates nitric oxide and causes endothelial damages and dysfunction. On the other hand, ANG-2 could drive focal inflammation by increasing expressions of pro-inflammatory cytokines and chemokines, which leads to leukocytes infiltration and eventually plaque formation. As shown in Fig. 1d, NXT significantly down-regulated serum ANG-2 level compared with MOD group (*P* < 0.05). These results emphasized the beneficial effects of NXT on regulating blood pressures with anti-inflammatory and vascular protective actions. Serum myocardial enzymes (α-HBDH, CK, CK-MB and LDH) and CRP levels were always evaluated as vital indicators of myocardial injuries. As shown in Fig. 1d, serum myocardial enzymes and CRP levels were elevated in the MOD group compared with the CON group (*P* < 0.01). NXT treatment showed significant protective effects on the cardiovascular system through the significant decreases of these parameters (*P* < 0.01).

These results, when taken together, provided us a comprehensive insight into the effects of NXT on T2D with potent actions in alleviating insulin resistance, recovering lipid metabolic disruption, mitigating inflammations, lowering hypertension, improving vascular endothelial functions as well as reducing myocardial injuries.

### Regulation of NXT on gut microbiome in T2D

We next aimed to examine the effects of NXT on intestinal microbiota of T2D rats. Rat fecal samples were collected at the experimental endpoint for sequencing of 16s rRNA gene. After sequencing data analysis with QIIME 2 2020.2^31^, a total of 642 ASVs were determined (with 100% similarity).

**Figure 2:**
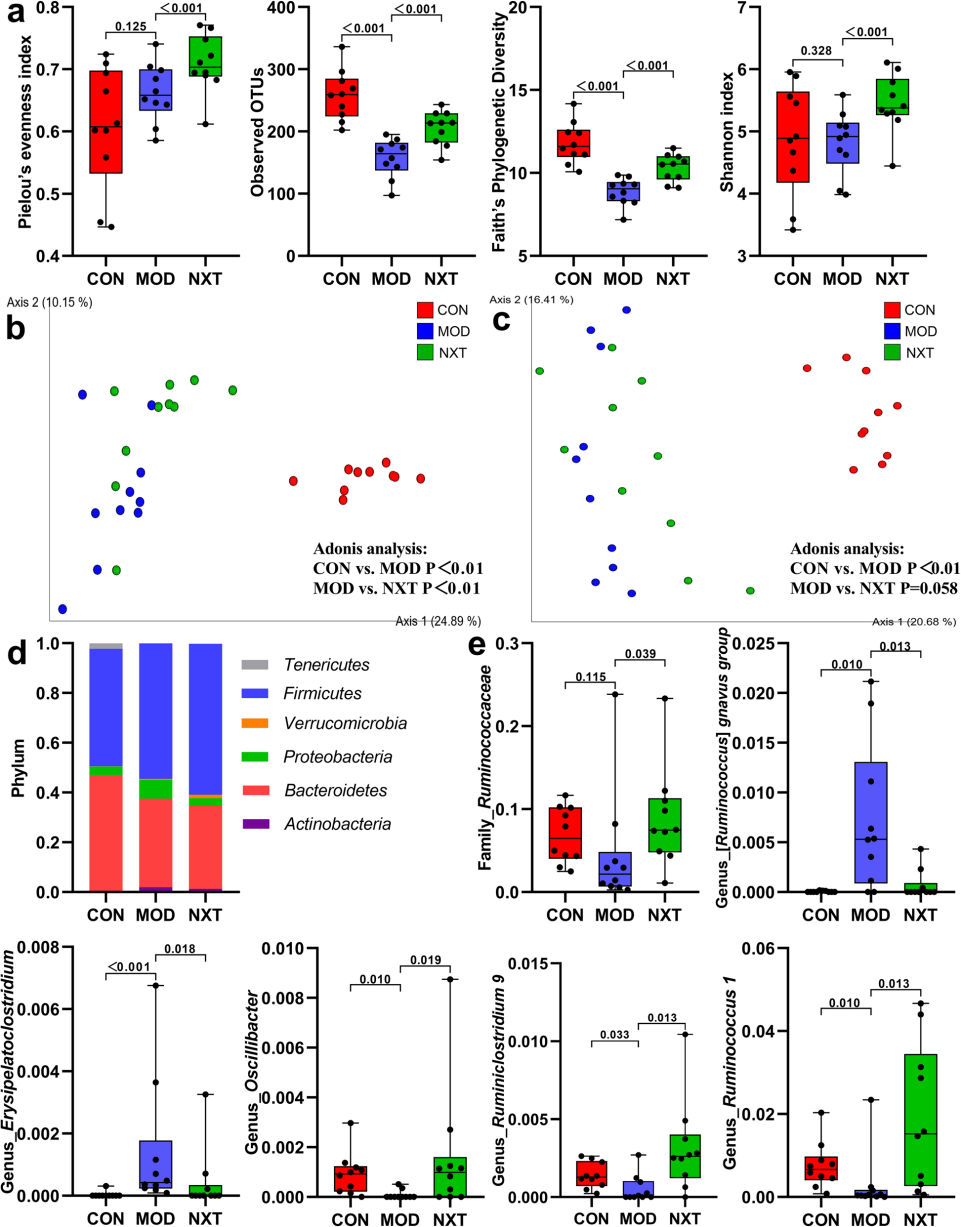
NXT regulated gut microbiota disturbance in T2D rats. (**a**) The α-diversity metrics (Pielou’s evenness index, observed OTUs, Faith’s phylogenetic diversity and Simpson index); (**b**) PCoA plots based on unweighted unifrac; (**c**) PCoA plots based on Bray–Curtis dissimilarity; (**d**) Compositions of gut microbiota at the phylum level; (**e**) Relative abundances of *Ruminococcaceae*, [*Ruminococcus*] *gnavus group*, *Erysipelatoclostridium*, *Oscillibacter*, *Ruminiclostridium 9* and *Ruminococcus 1*.Data were presented as mean ± SD (n = 10 in each group). CON, control group; MOD, model group; NXT, NXT-treated group. A value of P.adj < 0.05 was considered statistically significant by Mann–Whitney U test.

The Pielou’s evenness index, observed OTUs, Faith’s phylogenetic diversity and Simpson index were calculated to estimate the α-diversity metrics. As shown in Fig. 2a, observed OTUs and Faith’s phylogenetic diversity suggested a significant decrease in microbial richness in fecal samples of T2D rats compared with control rats (P.adj < 0.01), and Pielou’s evenness index, observed OTUs, Faith’s phylogenetic diversity and Simpson index were significantly regulated by NXT treatment (P.adj < 0.01). PCoA plots based on unweighted unifrac (Fig. 2b) and Bray–Curtis dissimilarity (Fig. 2c) were carried out to compare β-diversity of gut microbiota among tested groups. Besides, Adonis analysis based on distance matrix was also performed to determine significant difference in β-diversity of gut microbiota, which showed significant difference between CON and MOD in both unweighted unifrac and Bray–Curtis dissimilarity (Adonis *P* < 0.01) and significant difference between MOD and NXT only in unweighted unifrac (Adonis *P* < 0.01). In accord with literatures, our results indicated a significant gut microbial shift during the development of T2D, which highlighted the value of gut microbiota in diabetic pathogenesis. In addition, NXT partially recovered the richness and diversity of microbial community while had limited effects on β-diversity metrics of gut microbiota in T2D rats.

In addition, we further analyzed the relative abundances of differential bacteria in the gut microbiota at multiple levels. At the phylum level, phylum *Firmicutes* and *Bacteroidetes* were most abundantly presented which accounted for the majority of the population in all groups. T2D rats had a higher abundance of *Firmicutes* and a lower abundance of *Bacteroidetes* when compared with CON rats (Fig. 2d). Next, we investigated the microbial differences among groups at family and genus levels, respectively. We employed Boruta package to figure out feature bacteria among groups. Significant differences of feature bacteria were determined by Mann–Whitney U test. Figure 2e showed part of differential bacteria, and Table S1 and Table S2 presented the information of all selected differential bacteria at family and genus levels, respectively. At the family and genus levels, a wide variety of gut bacteria, including [*Ruminococcus*] *gnavus group*, *Erysipelatoclostridium*, *Oscillibacter*, *Ruminiclostridium 9* and *Ruminococcus 1*, were significantly different between healthy and T2D rats (P.adj < 0.05) (Fig. 2e, Table S1 and Table S2). Moreover, NXT could significantly restore the abnormal relative abundances of [*Ruminococcus*] *gnavus group*, *Erysipelatoclostridium*, *Oscillibacter*, *Ruminiclostridium 9* and *Ruminococcus 1* in T2D rats at genus level (P.adj < 0.05) (Figs. 2e).

### Global metabolic profiling of serum metabolites in T2D rats

**Figure 3:**
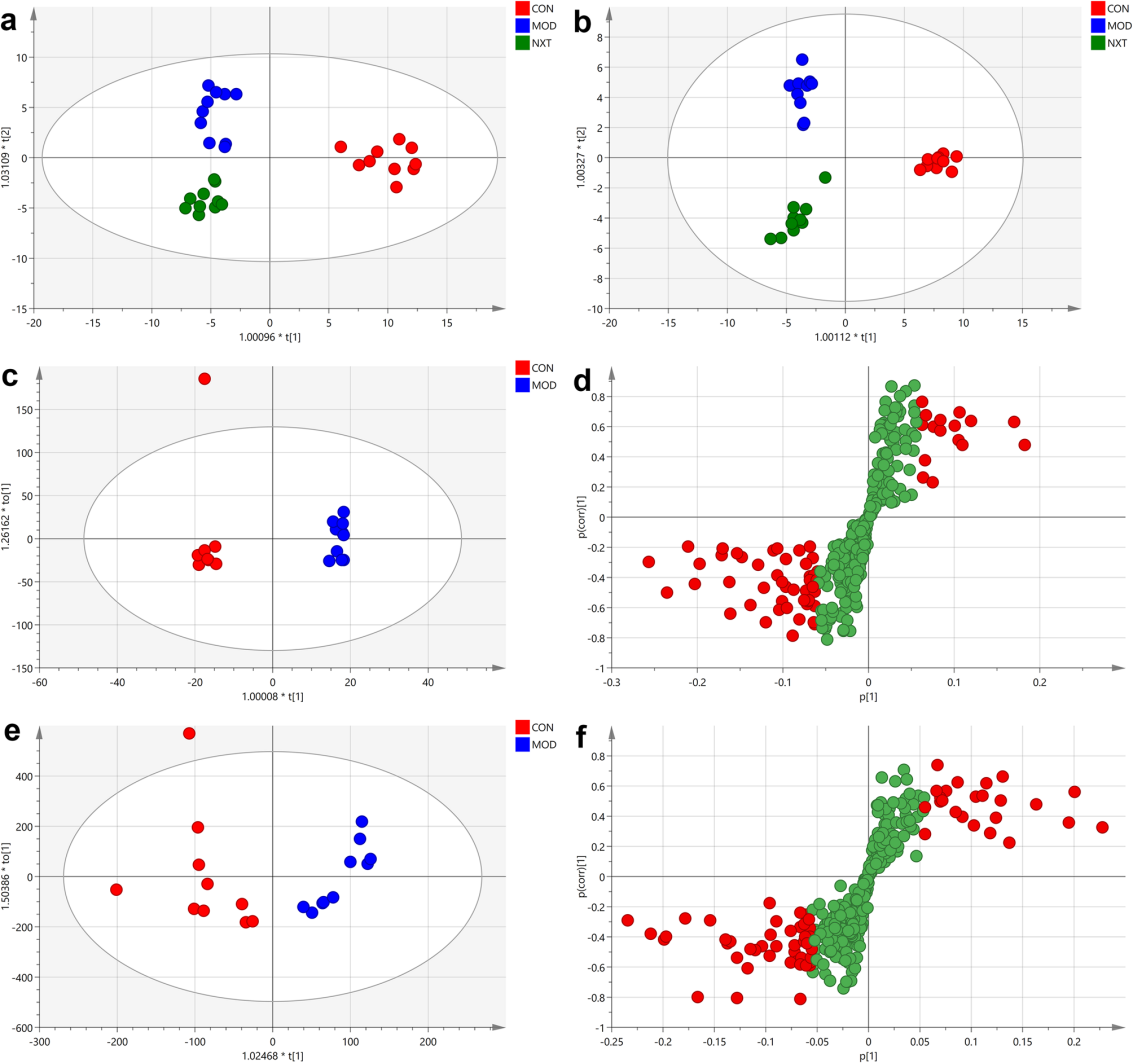
Multivariate analysis of identified peaks of serum metabolome. The 2D OPLS-DA score plots obtained from CON, MOD and NXT groups in negative (**a**) and positive (**b**) modes. The 2D OPLS-DA score plot (**c**) and S-plot (**d**) obtained from CON and MOD groups in the negative mode. The 2D OPLS-DA score plot (**e**) and S-plot (**f**) obtained from CON and MOD groups in the positive mode. CON, control group; MOD, model group; NXT, NXT-treated group.

Using UPLC-Q-TOF-MS/MS, we then applied untargeted metabolomics of serum samples to reveal the metabolic profiling among groups. MS data of serum samples were collected both in positive and negative ionization modes, and metabolites between 50 and 1,500 m/z were recorded and identified (Figure S1). During detection, pooled QC samples were inserted at 8 intervals to monitor system stability and reproducibility. The RSDs of the peak intensities and retention times of detected peaks of base peak chromatograms in positive and negative modes were calculated and more than 80% of RSDs were less than 30% for QC samples. After noise reduction and peak alignment and identification using PeakView® 2.2 and Markerview 1.2 software (AB Sciex, Foster City, USA), A total of 302 peaks in the negative mode and 354 peaks in the positive mode were identified for further analysis. Multivariate statistical analysis of identified peaks was applied to figure out differential metabolites among CON, MOD and NXT groups. In OPLS-DA analysis of CON, MOD and NXT groups, the score plots exhibited distinct populations among three groups (Fig. 3a, b) in both positive and negative modes, which suggested that T2D significantly reshaped the metabolic patterns of serum metabolites in rats. In order to better understand the T2D specific metabolic shifts, we also applied OPLS-DA analysis for the identified peaks of CON and MOD groups in both positive and negative modes (Fig. 3c, e). For OPLS-DA models obtained from CON and MOD groups, the cumulative R2X, R2Y and Q2 were 0.94, 0.984 and 0.879 in the negative mode and 0.911, 0.982 and 0.757 in the positive mode. As shown in Figure S2, the R2 Y-intercepts were 0.853 and 0.846 in negative and positive modes, respectively, and all R2 values and Q2 values to the left were lower than their original points to the right. The P values for the OPLS-DA models were less than 0.05 by analysis of variance (ANOVA) in the cross-validated residuals of a Y-variable, suggesting that the models were reliable. These results indicated that the OPLS-DA models were valid without overfitting. Based on the OPLS-DA models obtained from CON and MOD groups, the potential differential peaks between CON and MOD groups in negative and positive modes were picked out with VIP values > 1 (Figs. 3d and 3f). The potential differential peaks were further filtered out based on significance tests with *P* values < 0.05.

The chemical identification of these feature metabolites was achieved by local database as well as online databases including Chemspider (www.chemspider.com) and HMDB (www.hmdb.ca)^39^ according to mass fragmentation patterns and isotope peak ratios. A total of 45 potential differential biomarkers were identified (Table 1), among which 32 biomarkers were highly associated with the outcomes of NXT treatment.

**Table 1:** Potential biomarkers in serum samples of CON, MOD and NXT-treated groups.

No	Retention time (min)	Metabolite identification	Molecular formula	Ion form	Measured mass (Da)	Error (ppm)	Content (calibrated intensity)		
							CON	MOD	NXT
1	1.04	Alanine	C_3_H_7_NO_2_	[M − H]^−^	88.0404	0	0.38 ± 0.29	0.075 ± 0.07*	0.12 ± 0.08
2	1.07	Glutamine	C_5_H_10_N_2_O_3_	[M − H]^−^	145.0619	0	1.19 ± 0.97	0.51 ± 0.17*	0.68 ± 0.35
3	1.11	L-carnitine	C_7_H_15_NO_3_	[M + H]^+^	162.1119	3.7	83.65 ± 48.3	29.09 ± 5.22*	47.62 ± 20.26^#^
4	1.34	tyrosine	C_9_H_11_NO_3_	[M + H]^+^	182.0812	0	194.67 ± 85.33	123.44 ± 31.83*	191.13 ± 71.08^#^
5	5.05	Butyrylcarnitine	C_11_H_21_NO_4_	[M + H]^+^	232.1544	0.4	56 ± 24.92	19.11 ± 9.81*	22.02 ± 12.04
6	5.25	Tryptophan	C_11_H_12_N_2_O_2_	[M + H]^+^	205.0967	2.4	505.15 ± 238.83	191.08 ± 43.68*	305.34 ± 116.27^#^
7	5.29	Indoleacrylic acid	C_11_H_9_NO_2_	[M + H]^+^	188.0702	2.1	847.2 ± 424.09	342.04 ± 86.25*	541 ± 185.55^#^
8	5.79	Valerylcarnitine	C_12_H_23_NO_4_	[M + H]^+^	246.1695	2.0	20.88 ± 10.15	7.58 ± 3.38*	8.44 ± 4.65
9	7.08	11-Dehydrothromboxane B2	C_20_H_34_O_6_	[M + H]^+^	371.2428	0	2.29 ± 1.91	3.77 ± 1.17*	2.02 ± 1.54^#^
10	7.33	LPA (8:0/0:0)	C_11_H_23_O_7_P	[M − H]^−^	297.1058	17.2	2.38 ± 1.44	0.23 ± 0.30*	0.12 ± 0.13
11	7.68	3-Hydroxysebacic acid	C_10_H_18_O_5_	[M − H]^−^	217.1115	15.6	0.08 ± 0.08	0.29 ± 0.18*	0.15 ± 0.08^#^
12	8.01	Sulfolithocholylglycine	C_26_H_43_NO_7_S	[M − H]^−^	512.2748	11.9	7.34 ± 3.74	1.32 ± 0.77*	0.94 ± 0.82
13	8.56	Taurochenodeoxycholic acid	C_26_H_45_NO_6_S	[M − H]^−^	498.2969	14.9	0.38 ± 0.18	2.87 ± 1.70*	0.43 ± 0.27^#^
14	8.66	LPC (15:0)	C_23_H_48_NO_7_P	[M − H 20-H]^−^	462.2923	13.2	3.27 ± 2.64	0.59 ± 0.52*	1.25 ± 0.81^#^
15	9.22	Glycocholic acid	C_26_H_43_NO_6_	[M − H]^−^	464.3041	4.9	5.29 ± 3.65	10.29 ± 4.72*	3.51 ± 2.40^#^
16	9.25	20-hydroxy-leukotriene B4	C_20_H_32_O_5_	[M − H]^−^	351.2177	0	0.5 ± 0.61	1.12 ± 0.50*	0.92 ± 0.20
17	9.52	3-oxocholic acid	C_24_H_38_O_5_	[M − H]^−^	405.2662	3.9	0.59 ± 0.32	2.00 ± 2.04*	5.79 ± 5.78
18	9.52	Prostaglandin I2	C_20_H_32_O_5_	[M + H]^+^	353.2348	7.1	2.33 ± 0.99	21.68 ± 17.92*	7.66 ± 7.16^#^
19	9.53	3-oxo-4,6-choladienoic acid	C_24_H_34_O_3_	[M + H]^+^	371.2580	0.3	4.76 ± 1.89	5.75 ± 5.71	17.36 ± 14.88^#^
20	9.53	7alpha-hydroxy-3-oxochol-4-en-24-oic Acid	C_24_H_36_O_4_	[M + H]^+^	389.2687	0.3	10.77 ± 2.68	13.58 ± 13.57	41.34 ± 35.68^#^
21	9.7	Leukotriene E3	C_23_H_39_NO_5_S	[M − H]^−^	440.2518	9.5	0.24 ± 0.31	1.32 ± 1.25*	0.38 ± 0.31^#^
22	9.96	3-xix-7-hydrixychol-4-enoic acid	C_24_H_36_O_5_	[M + FA-H]^−^	449.2601	12.5	0.44 ± 0.24	0.06 ± 0.06*	0.21 ± 0.21^#^
23	10.01	Leukotriene B4	C_20_H_32_O_4_	[M − H]^−^	335.2243	4.5	0.2 ± 0.26	0.72 ± 0.51*	0.32 ± 0.08^#^
24	10.14	PC (18:0/22:6)	C_48_H_84_NO_8_P	[M + H]^+^	834.6083	9.1	12.38 ± 5.93	18.59 ± 19.26	53.1 ± 36.20^#^
25	10.45	Glycoursodeoxycholic acid	C_26_H_43_NO_5_	[M − H]^−^	448.3093	5.6	0.73 ± 0.36	1.29 ± 0.58*	0.46 ± 0.39^#^
26	10.86	Tetradecanoylcarnitine	C_21_H_41_NO_4_	[M + H]^+^	372.3108	0	7.56 ± 6.37	15.51 ± 9.83*	13.33 ± 10.65
27	10.94	Sphingosine	C_18_H_37_NO_2_	[M + H]^+^	300.2893	1.3	87.57 ± 57.32	73.87 ± 21.01	49.52 ± 24.98^#^
28	10.94	Oleamide	C_18_H_35_NO	[M + H]^+^	282.2784	2.5	61.96 ± 40.37	47.98 ± 12.74	31.83 ± 15.37^#^
29	11.09	13-HDoHE	C_22_H_34_O	[M − H]^−^	313.2505	10.2	0.27 ± 0.16	0.90 ± 0.67*	0.13 ± 0.05^#^
30	11.11	LysoPE (16:0/0:0)	C_21_H_44_NO_7_P	[M + Hac-H]^−^	512.3018	4.7	9.08 ± 4.59	1.78 ± 0.56*	3.16 ± 1.38^#^
31	11.19	18-hydroxycortisol	C_21_H_30_O_6_	[M − H]^−^	377.2022	13.8	0.19 ± 0.14	3.38 ± 1.96*	1.56 ± 0.81^#^
32	11.43	LysoPC (16:1)	C_24_H_48_NO_7_P	[M + H]^+^	494.3165	15.4	596.51 ± 372.68	187.44 ± 154.36*	170.91 ± 104.20
33	11.45	LysoPE (18:1/0:0)	C_23_H_46_NO_7_P	[M + Hac-H]^−^	538.3199	9.1	31.34 ± 21.52	3.56 ± 1.18*	6.42 ± 4.08^#^
34	11.65	Palmitoylcarnitine	C_23_H_45_NO_4_	[M + H]^+^	400.3421	0	35.72 ± 32.15	83.01 ± 51.25*	84.37 ± 55.44
35	12.57	Stearoylcarnitine	C_25_H_49_NO_4_	[M + H]^+^	428.3721	3.0	30.8 ± 15.10	346.93 ± 147.98*	98.02 ± 79.25^#^
36	12.6	Eicosapentaenoic acid	C_20_H_30_O_2_	[M + H]^+^	303.2308	3.6	483.22 ± 278.28	873.29 ± 337.36*	604.73 ± 178.10^#^
37	12.6	5-HPETE	C_20_H_32_O_4_	[M − H]^−^	335.2228	0	0.51 ± 0.68	1.19 ± 0.55*	0.61 ± 0.27^#^
38	12.6	DG(14:0/18:1/0:0)	C_35_H_66_O_5_	[M + K]^+^	605.4533	1.5	26.83 ± 21.49	130.88 ± 74.16*	76.36 ± 31.13^#^
39	12.81	LysoPC (16:0)	C_24_H_50_NO_7_P	[M + Hac-H]^−^	554.3521	10.5	17.53 ± 22.89	1.32 ± 0.43*	3.04 ± 2.46^#^
40	12.82	LysoPC (20:2)	C_28_H_54_NO_7_P	[M + H]^+^	548.3623	16.0	293.98 ± 164.82	108.42 ± 97.63*	97.81 ± 31.13
41	12.89	15S-HETrE	C_20_H_34_O_3_	[M − H]^−^	321.2406	9.0	1.99 ± 1.68	4.23 ± 2.31*	2.05 ± 0.63^#^
42	13.87	Linolenic acid	C_18_H_30_O_2_	[M − H]^−^	277.2183	3.6	1.59 ± 0.77	0.69 ± 0.30*	0.53 ± 0.21
43	14.13	Docosahexaenoic acid	C_22_H_32_O_2_	[M − H]^−^	327.2384	16.5	11.46 ± 6.55	14.04 ± 7.24	6.61 ± 1.94^#^
44	14.29	Arachidonic acid	C_20_H_32_O_2_	[M − H]^−^	303.2367	12.2	10.87 ± 3.89	19.59 ± 7.87*	12.76 ± 2.95^#^
45	15.66	Stearic acid	C_18_H_36_O_2_	[M − H]^−^	283.2686	15.2	1.05 ± 0.40	2.22 ± 0.79*	1.51 ± 0.22^#^

*P < 0.05 vs. CON group; ^#^*P* < 0.05 vs. MOD group.

Next, in order to provide a more comprehensive insights into the mechanisms of NXT, we performed pathway analysis of feature metabolites using MetaboAnalyst 4.0 (www.metaboanalyst.ca)^40^ and KEGG database (www.kegg.jp). In order to figure out the metabolic pathways highly associated with the outcomes of NXT treatment, the pathway analysis was performed using the module “Pathway Analysis” and the inputs were compound names of 32 biomarkers that were significantly regulated by NXT treatment listed in Table 1. Metabolic pathways closely related to NXT were figured out, including phenylalanine, tyrosine and tryptophan biosynthesis, arachidonic acid metabolism, glycerophospholipid metabolism, tryptophan metabolism, tyrosine metabolism, primary bile acid biosynthesis and sphingolipid metabolism with impact values of 0.50, 0.46, 0.18, 0.16, 0.14, 0.06 and 0.05, respectively (Table 2).

**Table 2:** Summary of metabolic pathway analysis of serum metabolome using MetaboAnalyst 4.0.

Pathway name	Match status	*P* value	− log(p)	Holm p	FDR	Impact
Phenylalanine, tyrosine and tryptophan biosynthesis	1/4	0.050	2.99	1	0.85	0.5
Arachidonic acid metabolism	5/36	0.000057	9.78	0.0046	0.0046	0.46
Glycerophospholipid metabolism	2/30	0.055	2.90	1	0.85	0.18
Tryptophan metabolism	1/41	0.42	0.88	1	1	0.16
Tyrosine metabolism	1/42	0.42	0.86	1	1	0.14
Primary bile acid biosynthesis	2/46	0.12	2.16	1	1	0.06
Sphingolipid metabolism	1/21	0.24	1.43	1	1	0.05
Biosynthesis of unsaturated fatty acids	4/42	0.0016	6.46	0.13	0.064	0
Ubiquinone and other terpenoid-quinone biosynthesis	1/3	0.038	3.27	1	0.85	0
Linoleic acid metabolism	1/5	0.063	2.77	1	0.85	0
Phenylalanine metabolism	1/9	0.11	2.21	1	1	0
alpha-Linolenic acid metabolism	1/9	0.11	2.21	1	1	0
Aminoacyl-tRNA biosynthesis	2/67	0.21	1.56	1	1	0
Fatty acid biosynthesis	1/43	0.43	0.84	1	1	0

### Correlations between microbial abundances and metabolic profiling

**Figure 4:**
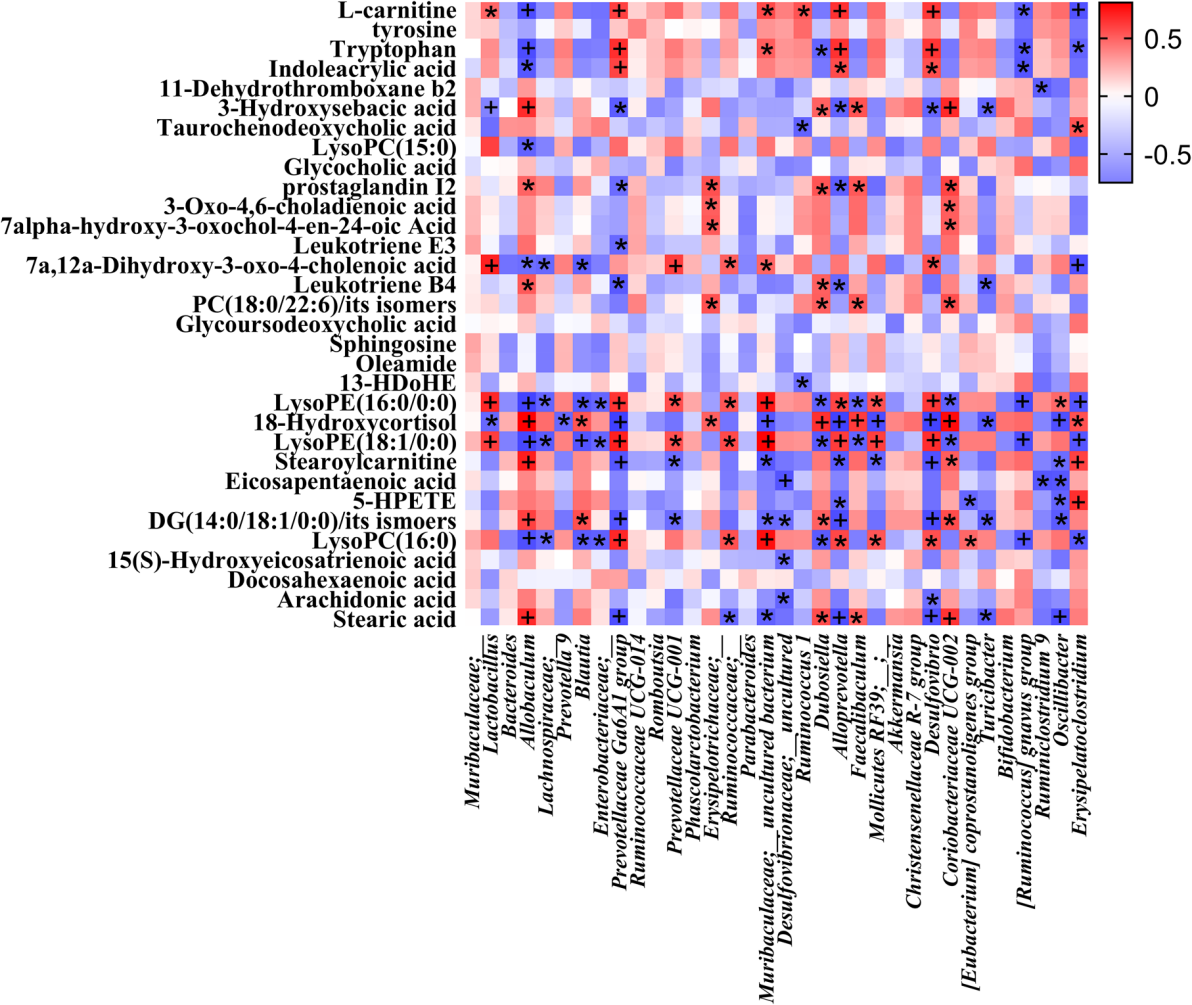
Correlation analysis of relative abundances of gut microbiota in the genus level and serum metabolite levels. *P.adj < 0.05; ^+^P.adj < 0.01.

Finally, to further dig out the correlations between serum metabolites and intestinal microbial communities, we conducted Spearman’s correlation analysis to reveal the potential relationships between microbial abundances at the genus level and serum metabolites. The coefficients of correlation between potential biomarkers highly associated with NXT treatment and relative abundances of gut microbiota at the genus level are presented in a heatmap (Fig. 4). Various metabolites could be well predicted by the relative abundances of gut microbiota significantly modulated by NXT treatment (P.adj < 0.05). Some of these metabolites are known as co-metabolites of certain strains of gut microbiota and could directly participate in microbial metabolism, such as L-carnitine, tryptophan, and indoleacrylic acid^18,41^.

## Discussion

In our study, a rat phenotype of T2D was established by 10-week HFD combined with a low-dose injection of STZ in the sixth week. HFD feeding induced insulin resistance and hyperlipidemia, and led obesity first in week 6 (*P* < 0.01). A low-dose of STZ injection was administered to cause pancreatic β-cell damage. The model rats then lost their weight with chronic and marked hyperglycemia after STZ injection and eventually, the body weight of the MOD group significantly continued to decrease compared with the CON group since week 11 (*P* < 0.01). Diabetes patients commonly suffer from abnormal and continuous body weight loss particularly when their blood glucose levels cannot be controlled validly^42^ and the abnormal body weight loss of T2D patients indicated increased glycemic variability which serves as a risk factor of diabetic complications^43^. It is inferred that the low body weight of T2D rats in this study was associated with pancreatic β-cell damage, intestinal inflammation and ectopic fat accumulation^44,45^. NXT treatment had a tendency of alleviating abnormal weight loss, suggesting that it might help blood glucose control. Effective control of the blood glucose level is crucial for the treatment of T2D patients^46^. The results of serum chemical analysis, OGTT and ITT suggested that NXT treatment could significantly regulate glucose metabolism in terms of lowering the blood glucose level and improving insulin resistance. Dyslipidemia is another characteristic of T2D, which also serves as an increasing risk of cardiovascular diseases^47^. NXT treatment significantly recovered the abnormal serum levels of TG, TC, HDL-C and FFAs in T2D. In recent years, a growing number of researches have indicated that low-grade inflammation plays a vital role in insulin resistance and pancreatic β-cells damage in the pathogenesis of T2D and also contributes to cardiovascular diseases^48^. The turbulences of serum cytokines (TNF-α, IL-1β, IL-4, and IL-6) in T2D rats were significantly altered by NXT treatment, suggesting that NXT could mitigate low-grade inflammation in T2D. As for diabetic complications, NXT treatment appeared to lower blood pressure according to SYS, DIA and MAP. The renin-angiotensin system plays critical roles in maintaining normal cardiovascular functions and ANG-2 is an important polypeptide for blood pressure regulation. A significant decrease of serum ANG-2 level contributed to the hypotensive effect of NXT treatment. Moreover, decreased levels of myocardial enzymes (α-HBDH, CK, CK-MB and LDH), CRP and ET-1 in serum implied less myocardial and endothelial injuries in T2D rats treated by NXT.

16s rRNA gene V4-V5 region amplification and sequencing were carried out to investigate the impact of NXT treatment on gut microbiota in T2D. Interestingly, significant imbalance of gut microbiota was observed in T2D rats when compared with healthy rats. Community diversity is crucial for ecosystem stability and efficiency and reduced diversity in gut microbiota was previously observed in various diseases such as T2D and cardiovascular diseases^49,50^. In this study, α-diversity metrics including Pielou’s evenness index, observed OTUs, Faith’s phylogenetic diversity and Shannon index were calculated to evaluate the community richness and evenness of gut microbiota. The observed OTUs and Faith’s phylogenetic diversity was significantly decreased in T2D rats when compared with healthy rats, which indicated that T2D rats had a smaller number of species in gut microbiota, while there was no significant difference in community evenness of gut microbiota in T2D and healthy rats according to the Pielou’s evenness index and Shannon index. Notably, NXT treatment significantly enhanced community diversity of gut microbiota in T2D rats according to all calculated α-diversity metrics. This is likely a result of a large number of components in NXT such as dietary fibers, which served as prebiotics that many gut bacteria derive energy from^21,23^. As for β-diversity metrics, unweighted unifrac and Bray–Curtis dissimilarity were used to evaluate the differences in gut microbiota compositions among tested groups based on different standards. PcoA analysis and Adonis analysis based on unweighted unifrac and Bray–Curtis dissimilarity showed a significant difference of gut microbiota between T2D and healthy rats. In addition, NXT treatment could affect the compositions of gut microbiota to a certain degree in view of unweighted unifrac. At the phylum level, the T2D rats had a relatively high abundance of *Firmicutes* and a relatively low abundance of *Bacteroidetes* compared with the healthy rats. The shifts at the phylum level was also found in obesity, T2D and coronary artery disease patients and rats in previous studies, indicating that changes at the phylum level may be associated with various health problems^51,52^. At the genus and family levels, as shown in Fig. 2, Table S1 and Table S2, the relative abundances of diverse intestinal bacteria were significantly shifted in T2D rats compared with healthy rats and NXT treatment could alter the relative abundances of certain intestinal bacteria, such as *Deferribacteraceae*, *Helicobacteraceae* and *Ruminococcaceae*. Among these, NXT treatment could restore the changed relative abundances of *Ruminococcaceae* as well as multiple genera belonging to *Ruminococcaceae* like *Ruminococcus 1* and *Ruminiclostridium 9*. *Ruminococcaceae* is a main family with a relatively high abundance in gut microbiota which is believed to produce short-chain fatty acids^53,54^, and short-chain fatty acids could serve as an energy substrate to colonocytes and mitigate inflammation in the intestinal tract^55^. In a clinal study participated by 617 middle-aged women, two OTUs belonging to *Ruminococcaceae* were negatively correlated with carotid-femoral pulse wave velocity that is an important indicator in cardiovascular diseases^56^. Besides, NXT group had a lower relative abundance of *Erysipelatoclostridium* compared with MOD group, which was associated with some diseases like kidney stones^57^. Up to now, most of literatures as well as this study used 16s rRNA gene sequencing and focused on the compositions and statistical correlations of gut microbiota^58^. However, gut microbiota is increasingly recognized to affect host health through its unique metabolic functions and even different strains of one species of gut microbiota might have different effects on health^21,59^. Although some kinds of gut microbiota were observed to be modulated by NXT treatment in this study, the direct roles of gut microbiota in mechanisms of NXT treatment are yet unclear and the results could provide a scientific basis for further experiments.

**Figure 5:**
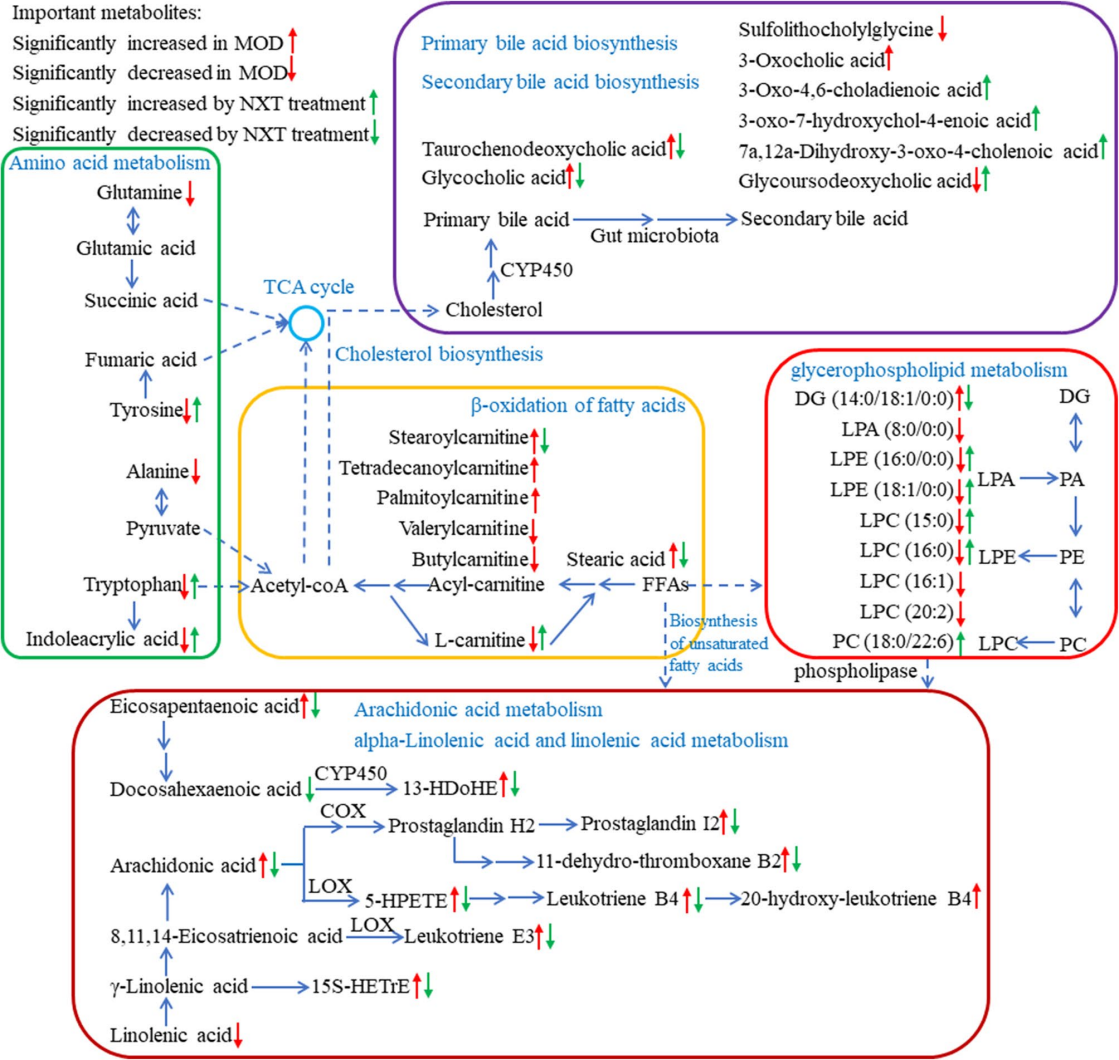
Proposed Metabolic networks of NXT on T2D.

Multivariate statistical analysis indicated that the metabolic profiles of serum samples were distinctly different between CON and MOD groups. It was clear that the metabolic turbulence in T2D rats was partly restored by NXT treatment. To investigate the potential mechanisms of NXT, important differential metabolites are summarized in Fig. 5 using metabolic pathways reported in KEGG database (www.kegg.jp), SMPDB database (smpdb.ca)^60^ and the published literatures^61^ with Metaboanalyst 4.0 (www.metaboanalyst.ca)^40^. We found that NXT treatment in T2D rats was mainly associated with lipid metabolism.

Eicosapentaenoic acid, docosahexaenoic acid and linolenic acid are members of the group of essential polyunsaturated fatty acids (PUFAs) called omega-3 fatty acids, which are involved in alpha-linolenic acid and linolenic acid metabolism in T2D. It is generally considered that omega-3 fatty acids play an active therapeutic role in metabolic diseases, including lowering serum lipid and glucose level and reducing risks of cardiovascular disorders^62^. The benefit effects of omega-3 fatty acids and their oxidation products are partly due to modulation of the gene expression of several pathway such as energy metabolism and arachidonic acid metabolism^63^. Among them, 15S-HETrE suppresses COX-2 over expression and/or prostaglandin E2 biosynthesis, and promotes the expression of PPARγ expression which is critical in the regulation of insulin sensitivity, adipogenesis and blood pressure^63,64^. in vivo, arachidonic acid is an important 20-carbon PUFA synthesized from linoleic acid and mediates multiple physiological functions either directly or upon its conversion into eicosanoids^65^. Eicosanoids are extremely potent, which are able to cause profound physiological effects at very dilute concentrations. Among eicosanoids, leukotrienes, mainly derived from arachidonic acid via LOX, serve as inflammatory mediators and regulate inflammatory cytokine production^66^. Prostaglandins and thromboxanes are both produced from arachidonic acid via COX and work as antagonists in platelet aggregation, thrombosis and other processes^67^. Arachidonic acid and several PUFAs are constituents of animal phosphatides and phospholipase can catalyze the hydrolysis of membrane glycerophospholipids to liberate them to initiate the arachidonate cascade and eicosanoid production^68^. As shown in Fig. 5, several PUFAs and their oxidation products increased in MOD group which resulted in the inflammatory and hypercoagulable state at the diabetic condition, and NXT treatment significantly regulated this turbulence.

As listed in Table 1, 3-hydroxysebacic acid has been regarded as an indicator of β-oxidation of fatty acids in previous studies^69^, and the level of 3-hydroxysebacic acid was elevated in MOD group, suggesting suppression of β-oxidation of fatty acids in T2D rats. We also found that the levels of long chain fatty acid (stearic acid) and long and medium chain acylcarnitines (stearoylcarnitine, tetradecenoylcarnitine and palmitoylcarnitine) were increased in MOD group compared with the CON group, whereas the levels of L-carnitine and short chain acylcarnitines (valerylcarnitine and butylcarnitine) were decreased. L-carnitine is essential for long and medium chain fatty acids to transport across mitochondrial membranes for β-oxidation. Notably, the metabolism by gut microbiota of dietary L-carnitine can produce TMAO and accelerate atherosclerosis in *vivo*^18^, and as shown in Fig. 5, the level of L-carnitine was well predicted by the abundances of several microbial genus. The significant effect of NXT treatment on the L-carnitine, stearic acid and stearoylcarnitine levels suggests that NXT could possibly promote β-oxidation of fatty acids to help blood lipid and glucose control and this effect might be associated with the modulation of gut microbiota.

Lysophospholipids, including LPCs, LPEs and LPAs, are major serum phosphatides and has been recognized as an important cell signaling molecules associated with cardiovascular and metabolic diseases^70,71^. Lysophospholipids are formed by hydrolysis of glycerophospholipids in glycerophospholipids metabolism pathway. In this study, a series of lysophospholipids were significantly decreased in MOD group compared with CON group, while were restored after NXT treatment. There were increasing evidences that lysophospholipids are involved in energy metabolism, inflammation and endothelial injury^70–72^. However, the mode of actions of lysophospholipids is not yet well understood.

Bile acids are steroids primarily synthesized from cholesterol via CYP450 and have the general functions of regulating fat absorption, cholesterol excretion and homeostasis of triglycerides and glucose. In recent years, bile acids have also been believed to act as hormones regulating various metabolic processes. Importantly, secondary bile acids are synthesized from primary bile acids by the gut microbiota. Gut microbiota is capable of regulating the bile acid pool and bile acid receptors in the intestinal tract to trigger various metabolic axes and alter host metabolism. Bile acids, in turn, can also affect the compositions of the gut microbiota^73^. Here, the higher levels of primary bile acids were observed in MOD group compared with CON group, which was reported to be a metabolic indicator of abnormal lipid metabolism in T2D^72^. Our results indicated that NXT could regulate cholesterol catabolism by reducing the levels of serum primary bile acids. In addition, the levels of several secondary bile acids were abnormal in T2D rats and altered by NXT treatment, which might be associated with the change of gut microbiota.

In addition to the above metabolites, other differential metabolites associated with amino acid metabolism (triose, tryptophan and indoleacrylic acid), steroid hormone biosynthesis (18-hydroxycortisol) and sphingosine metabolism (sphingosine) were also found to be regulated by NXT treatment in this work. It indicated that these metabiotic pathways directly regulated by NXT treatment may contribute to its pharmacological effects as well.

Taken together, based on the clinical practice and TCM theory, we determined that the treatment of NXT in T2D rats could improve hyperglycemia and hyperlipidemia, mitigate inflammation, lower blood pressure and protect against cardiovascular injuries. An integrative serum metabolomics and gut microbiome study further provided a global understand of the mechanisms of NXT. We found that NXT treatment was likely to improve multiple metabiotic pathways and gut microbiota to maintain its multipronged therapeutic effects on T2D and its complications. Our study indicates that NXT treatment might be an alternative strategy to current interventions for T2D and its complications and could offer additional benefits to patients. In view of the complex compositions of NXT, it is inferred that the direct effects on metabolic pathways and indirect effects on gut microbiota of NXT treatment together contributed to its pharmacological actions. Furthermore, several metabolites could be well predicted by the gut microbiota in this work, revealing underlying relationships between gut microbiota and host metabolism. However, the current studies are still limited and the direct relationships between gut microbiota and host metabolism need further investigations.

## Supplementary information

Supplementary file1 (DOCX 261 kb)

Supplementary file2 (XLSX 44 kb)

## Data Availability

All sequencing data can be viewed at NODE (https://www.biosino.org/node) by pasting the accession No. OEP000457 into the text search box or through the URL: https://www.biosino.org/node/project/detail/OEP000457. Raw data of pharmacological and metabolomics experiments were provided as a supplementary file (Dataset.xlsx). Other datasets used and/or analyzed during the current study are available from the corresponding author on reasonable request.
