# Supplementary material for: Integrated metabolomics and gut microbiome to the effects and mechanisms of naoxintong capsule on type 2 diabetes in rats
Source: Sci Rep. 2020 Jul 2;10:10829. doi: 10.1038/s41598-020-67362-2 (PMC7331749; doi:10.1038/s41598-020-67362-2)
Supplement: Supplementary file 1 — Supplementary file1 (DOCX 261 kb) [file 41598_2020_67362_MOESM1_ESM.docx]

**Integrated Metabolomics and Gut Microbiome to the Effects and Mechanisms of Naoxintong Capsule on Type 2 Diabetes in Rats**

Zenghao Yan^1^, Hao Wu^1*^, Haokui Zhou^2^, Shuo Chen^2^, Yan He^1^, Weijian Zhang^1^, Taobin Chen^1^, Hongliang Yao^3^, Weiwei Su^1^

^1^Guangdong Engineering & Technology Research Center for Quality and Efficacy Reevaluation of Post-market Traditional Chinese Medicine, Guangdong Key Laboratory of Plant Resources, State Key Laboratory of Biocontrol, School of Life Sciences, Sun Yat-sen University, Guangzhou, 510275, P. R. China

^2^Institute of Synthetic Biology, Shenzhen Institutes of Advanced Technology, Chinese Academy of Sciences, Shenzhen, 518055, P. R. China

^3^Guangdong Key Laboratory of Animal Conservation and Resource Utilization, Guangdong Public Laboratory of Wild Animal Conservation and Utilization, Drug Synthesis and Evaluation Center, Guangdong Institute of Applied Biological Resources, Guangdong, 510260, P. R. China

^*^ Corresponding author: Dr. Hao Wu, wuhao8@mail.sysu.edu.cn

Supplementary Materials

a

b

**Figure S1** typical UPLC-Q-TOF-MS/MS TIC chromatograms. TIC chromatograms of the serum QC sample in the negative ionization mode (a) and the positive ionization mode (b).

**
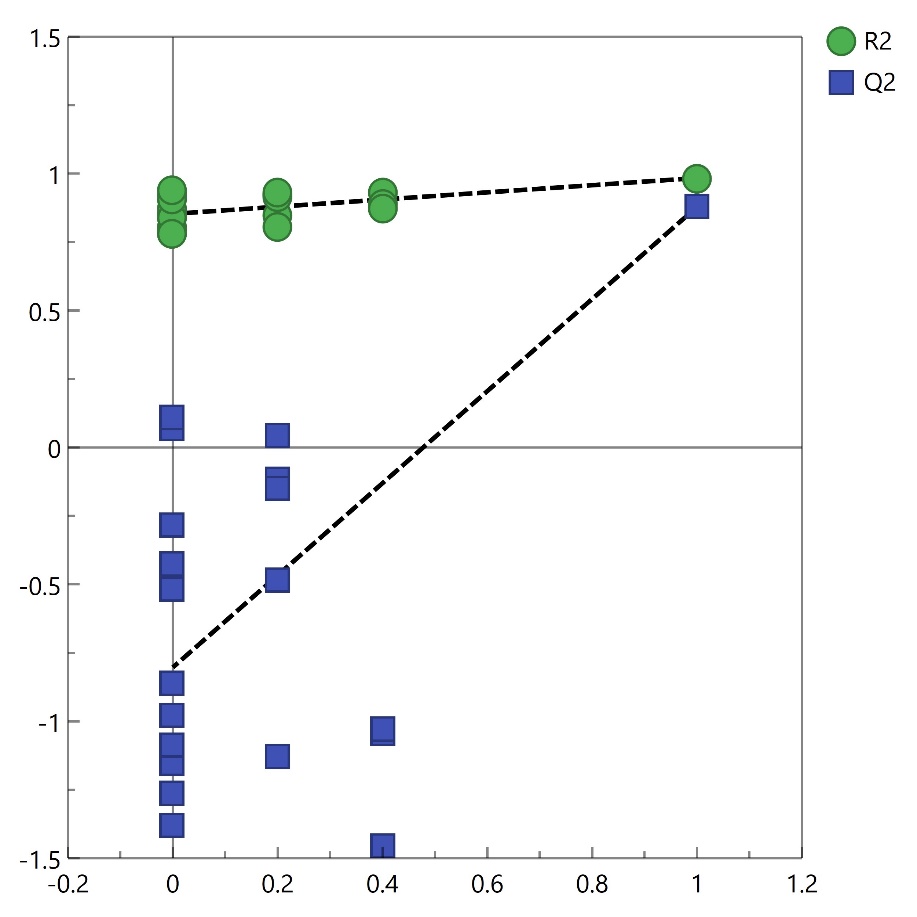
**
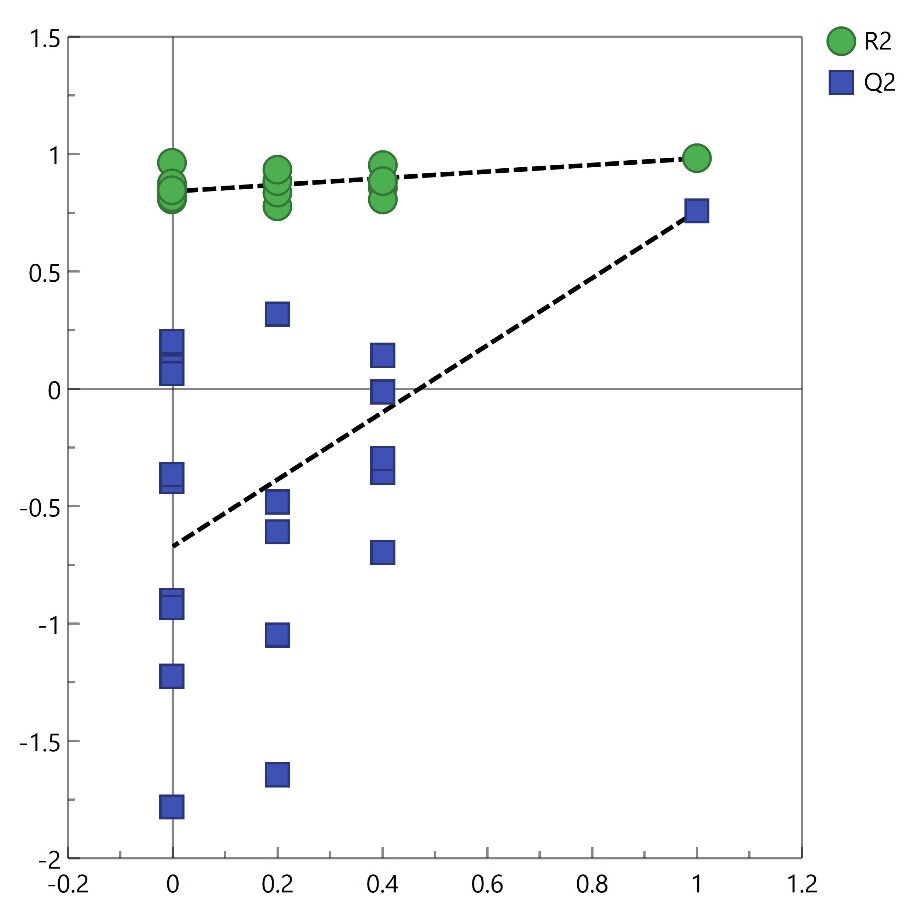


b

a

**Figure S2** Validation plots for OPLS-DA models from 20 permutation tests. (a) OPLS-DA model obtained from CON and MOD groups in the negative mode; (b) OPLS-DA model obtained from CON and MOD groups in the positive mode.

**Table S1** differential bacteria between CON and MOD or MOD and NXT at the family level. *P.adj < 0.05 vs. CON; **P.adj < 0.01 vs. CON; #P.adj < 0.05 vs. MOD.

| Taxonomy | Relative abundance | | |
| --- | --- | --- | --- |
|  | CON | MOD | NXT |
| *D_0__Bacteria; D_1__Deferribacteres; D_2__Deferribacteres; D_3__Deferribacterales; D_4__Deferribacteraceae* | 7.40E-05±2.34E-04 | 2.64E-05±5.68E-05 | 3.74E-04±5.00E-04# |
| *D_0__Bacteria; D_1__Epsilonbacteraeota; D_2__Campylobacteria; D_3__Campylobacterales; D_4__Helicobacteraceae* | 1.92E-04±2.61E-04 | 4.03E-04±4.32E-04 | 1.40E-03±7.41E-04# |
| *D_0__Bacteria; D_1__Firmicutes; D_2__Clostridia; D_3__Clostridiales; D_4__Ruminococcaceae* | 6.85E-02±3.38E-02 | 4.56E-02±7.17E-02 | 8.89E-02±6.04E-02# |
| *D_0__Bacteria; D_1__Proteobacteria; D_2__Alphaproteobacteria; D_3__Rhodospirillales; D_4__uncultured* | 0 | 0 | 6.65E-05±8.82E-05# |
| *D_0__Bacteria; D_1__Actinobacteria; D_2__Coriobacteriia; D_3__Coriobacteriales; D_4__Atopobiaceae* | 5.02E-04±4.00E-04 | 1.01E-02±9.94E-03** | 8.34E-03±6.97E-03 |
| *D_0__Bacteria; D_1__Actinobacteria; D_2__Coriobacteriia; D_3__Coriobacteriales; D_4__Eggerthellaceae* | 3.57E-04±3.22E-04 | 1.20E-03±7.80E-04** | 1.07E-03±6.73E-04 |
| *D_0__Bacteria; D_1__Bacteroidetes; D_2__Bacteroidia; D_3__Bacteroidales; D_4__Prevotellaceae* | 2.28E-01±9.26E-02 | 1.45E-02±1.47E-02** | 2.59E-02±2.97E-02 |
| *D_0__Bacteria; D_1__Bacteroidetes; D_2__Bacteroidia; D_3__Bacteroidales; D_4__Rikenellaceae* | 2.42E-03±1.80E-03 | 1.02E-04±2.01E-04** | 8.15E-05±1.09E-04 |
| *D_0__Bacteria; D_1__Elusimicrobia; D_2__Elusimicrobia; D_3__Elusimicrobiales; D_4__Elusimicrobiaceae* | 1.29E-04±1.78E-04 | 0* | 0 |
| *D_0__Bacteria; D_1__Firmicutes; D_2__Bacilli; D_3__Lactobacillales; D_4__Lactobacillaceae* | 2.76E-01±1.70E-01 | 3.90E-02±3.62E-02** | 8.20E-02±1.01E-01 |
| *D_0__Bacteria; D_1__Firmicutes; D_2__Clostridia; D_3__Clostridiales; D_4__Family XIII* | 2.25E-03±1.04E-03 | 5.82E-04±5.95E-04** | 1.22E-03±7.00E-04 |
| *D_0__Bacteria; D_1__Firmicutes; D_2__Clostridia; D_3__Clostridiales; D_4__Lachnospiraceae±* | 4.21E-02±2.26E-02 | 1.53E-01±5.18E-02** | 2.20E-01±9.92E-02 |
| *D_0__Bacteria; D_1__Firmicutes; D_2__Clostridia; D_3__Clostridiales; D_4__Peptococcaceae* | 7.08E-04±7.26E-04 | 1.21E-04±2.63E-04** | 1.21E-04±1.43E-04 |
| *D_0__Bacteria; D_1__Firmicutes; D_2__Erysipelotrichia; D_3__Erysipelotrichales; D_4__Erysipelotrichaceae* | 1.48E-02±1.51E-02 | 2.70E-01±1.82E-01** | 1.82E-01±9.96E-02 |
| *D_0__Bacteria; D_1__Proteobacteria; D_2__Deltaproteobacteria; D_3__Desulfovibrionales; D_4__Desulfovibrionaceae* | 2.88E-02±2.81E-02 | 6.67E-03±1.39E-02** | 1.22E-02±9.94E-03 |
| *D_0__Bacteria; D_1__Proteobacteria; D_2__Gammaproteobacteria; D_3__Enterobacteriales; D_4__Enterobacteriaceae* | 4.05E-03±3.38E-03 | 6.49E-02±1.00E-01* | 1.55E-02±2.29E-02 |
| *D_0__Bacteria; D_1__Tenericutes; D_2__Mollicutes; D_3__Mollicutes RF39; __* | 2.03E-02±2.10E-02 | 2.12E-05±4.46E-05** | 1.36E-05±4.29E-05 |

**Table S2** differential bacteria between CON and MOD or MOD and NXT at the genus level. *P.adj < 0.05 vs. CON; **P.adj < 0.01 vs. CON; #P.adj < 0.05 vs. MOD; ##P.adj < 0.01 vs. MOD.

| Taxonomy | Relative abundance | | |
| --- | --- | --- | --- |
|  | CON | MOD | NXT |
| *D_0__Bacteria; D_1__Bacteroidetes; D_2__Bacteroidia; D_3__Bacteroidales; D_4__Muribaculaceae; D_5__uncultured Bacteroidales bacterium* | 0 | 4.39E-06±1.39E-05 | 3.51E-03±4.68E-03## |
| *D_0__Bacteria; D_1__Bacteroidetes; D_2__Bacteroidia; D_3__Bacteroidales; D_4__Prevotellaceae; D_5__Prevotellaceae NK3B31 group* | 1.22E-03±1.27E-03 | 2.33E-04±3.19E-04 | 3.24E-03±3.53E-03# |
| *D_0__Bacteria; D_1__Deferribacteres; D_2__Deferribacteres; D_3__Deferribacterales; D_4__Deferribacteraceae; D_5__Mucispirillum* | 7.40E-05±2.34E-04 | 2.64E-05±5.68E-05 | 3.74E-04±5.00E-04# |
| *D_0__Bacteria; D_1__Epsilonbacteraeota; D_2__Campylobacteria; D_3__Campylobacterales; D_4__Helicobacteraceae; D_5__Helicobacter* | 1.92E-04±2.61E-04 | 4.03E-04±4.32E-04 | 1.40E-03±7.41E-04# |
| *D_0__Bacteria; D_1__Firmicutes; D_2__Clostridia; D_3__Clostridiales; D_4__Lachnospiraceae; D_5__[Ruminococcus] gnavus group* | 4.28E-05±7.10E-05 | 7.29E-03±7.53E-03** | 7.06E-04±1.46E-03# |
| *D_0__Bacteria; D_1__Firmicutes; D_2__Clostridia; D_3__Clostridiales; D_4__Ruminococcaceae; D_5__Oscillibacter* | 9.27E-04±8.60E-04 | 8.66E-05±1.87E-04** | 1.61E-03±2.64E-03# |
| *D_0__Bacteria; D_1__Firmicutes; D_2__Clostridia; D_3__Clostridiales; D_4__Ruminococcaceae; D_5__Ruminiclostridium 9* | 1.40E-03±8.27E-04 | 5.26E-04±8.87E-04* | 3.17E-03±2.92E-03# |
| *D_0__Bacteria; D_1__Firmicutes; D_2__Clostridia; D_3__Clostridiales; D_4__Ruminococcaceae; D_5__Ruminococcaceae UCG-010* | 0 | 0 | 1.93E-04±2.02E-04# |
| *D_0__Bacteria; D_1__Firmicutes; D_2__Clostridia; D_3__Clostridiales; D_4__Ruminococcaceae; D_5__Ruminococcaceae UCG-014* | 1.24E-02±9.40E-03 | 2.62E-02±6.63E-02 | 3.66E-02±4.34E-02# |
| *D_0__Bacteria; D_1__Firmicutes; D_2__Clostridia; D_3__Clostridiales; D_4__Ruminococcaceae; D_5__Ruminococcus 1* | 7.56E-03±5.58E-03 | 2.96E-03±7.23E-03* | 1.91E-02±1.75E-02# |
| *D_0__Bacteria; D_1__Firmicutes; D_2__Erysipelotrichia; D_3__Erysipelotrichales; D_4__Erysipelotrichaceae; D_5__Erysipelatoclostridium* | 3.10E-05±9.81E-05 | 1.40E-03±2.16E-03** | 4.18E-04±1.02E-03# |
| *D_0__Bacteria; D_1__Actinobacteria; D_2__Coriobacteriia; D_3__Coriobacteriales; D_4__Atopobiaceae; D_5__Coriobacteriaceae UCG-002* | 4.89E-04±4.06E-04 | 9.38E-03±9.54E-03** | 7.64E-03±6.54E-03 |
| *D_0__Bacteria; D_1__Actinobacteria; D_2__Coriobacteriia; D_3__Coriobacteriales; D_4__Atopobiaceae; D_5__Olsenella* | 1.32E-05±4.17E-05 | 6.84E-04±5.51E-04** | 7.02E-04±6.03E-04 |
| *D_0__Bacteria; D_1__Actinobacteria; D_2__Coriobacteriia; D_3__Coriobacteriales; D_4__Eggerthellaceae; D_5__Adlercreutzia* | 5.14E-05±8.62E-05 | 7.81E-04±5.42E-04** | 3.85E-04±2.80E-04 |
| *D_0__Bacteria; D_1__Bacteroidetes; D_2__Bacteroidia; D_3__Bacteroidales; D_4__Muribaculaceae; D_5__Muribaculum* | 4.87E-03±2.25E-03 | 8.20E-04±1.08E-03** | 1.25E-03±1.29E-03 |
| *D_0__Bacteria; D_1__Bacteroidetes; D_2__Bacteroidia; D_3__Bacteroidales; D_4__Muribaculaceae; D_5__uncultured bacterium* | 1.99E-02±8.05E-03 | 4.72E-03±3.58E-03** | 7.48E-03±6.20E-03 |
| *D_0__Bacteria; D_1__Bacteroidetes; D_2__Bacteroidia; D_3__Bacteroidales; D_4__Prevotellaceae; D_5__Alloprevotella* | 2.27E-02±3.09E-02 | 9.55E-04±1.33E-03** | 7.69E-04±6.92E-04 |
| *D_0__Bacteria; D_1__Bacteroidetes; D_2__Bacteroidia; D_3__Bacteroidales; D_4__Prevotellaceae; D_5__Prevotellaceae Ga6A1 group* | 7.90E-02±6.63E-02 | 4.23E-04±2.12E-04** | 4.40E-04±1.80E-04 |
| *D_0__Bacteria; D_1__Bacteroidetes; D_2__Bacteroidia; D_3__Bacteroidales; D_4__Rikenellaceae; D_5__Alistipes* | 2.26E-03±1.74E-03 | 1.02E-04±2.01E-04** | 6.97E-05±9.58E-05 |
| *D_0__Bacteria; D_1__Firmicutes; D_2__Bacilli; D_3__Lactobacillales; D_4__Lactobacillaceae; D_5__Lactobacillus* | 2.76E-01±1.70E-01 | 3.90E-02±3.62E-02** | 8.20E-02±1.01E-01 |
| *D_0__Bacteria; D_1__Firmicutes; D_2__Clostridia; D_3__Clostridiales; D_4__Family XIII; __* | 1.88E-03±9.50E-04 | 3.97E-04±4.64E-04** | 1.00E-03±5.92E-04 |
| *D_0__Bacteria; D_1__Firmicutes; D_2__Clostridia; D_3__Clostridiales; D_4__Lachnospiraceae; D_5__Blautia* | 3.91E-03±2.25E-03 | 4.05E-02±2.32E-02** | 4.85E-02±2.43E-02 |
| *D_0__Bacteria; D_1__Firmicutes; D_2__Clostridia; D_3__Clostridiales; D_4__Lachnospiraceae; D_5__Fusicatenibacter* | 1.06E-05±3.34E-05 | 2.03E-03±1.61E-03** | 2.13E-03±3.69E-03 |
| *D_0__Bacteria; D_1__Firmicutes; D_2__Clostridia; D_3__Clostridiales; D_4__Lachnospiraceae; D_5__Lachnoclostridium* | 6.69E-05±1.24E-04 | 2.71E-03±3.47E-03** | 2.20E-03±3.16E-03 |
| *D_0__Bacteria; D_1__Firmicutes; D_2__Clostridia; D_3__Clostridiales; D_4__Lachnospiraceae; D_5__Lachnospiraceae NK4A136 group* | 4.23E-03±3.72E-03 | 3.40E-05±5.58E-05** | 1.42E-04±2.37E-04 |
| *D_0__Bacteria; D_1__Firmicutes; D_2__Clostridia; D_3__Clostridiales; D_4__Lachnospiraceae; D_5__[Eubacterium] xylanophilum group* | 1.06E-03±8.47E-04 | 1.07E-05±3.40E-05** | 1.94E-04±3.91E-04 |
| *D_0__Bacteria; D_1__Firmicutes; D_2__Clostridia; D_3__Clostridiales; D_4__Lachnospiraceae; D_5__uncultured* | 2.33E-05±7.36E-05 | 5.65E-04±3.77E-04** | 7.49E-04±1.01E-03 |
| *D_0__Bacteria; D_1__Firmicutes; D_2__Clostridia; D_3__Clostridiales; D_4__Lachnospiraceae; __* | 3.08E-02±1.84E-02 | 9.65E-02±3.29E-02** | 1.57E-01±8.17E-02 |
| *D_0__Bacteria; D_1__Firmicutes; D_2__Clostridia; D_3__Clostridiales; D_4__Ruminococcaceae; D_5__Butyricicoccus* | 1.55E-05±4.91E-05 | 4.83E-04±3.91E-04** | 2.20E-04±2.65E-04 |
| *D_0__Bacteria; D_1__Firmicutes; D_2__Clostridia; D_3__Clostridiales; D_4__Ruminococcaceae; D_5__Flavonifractor* | 8.97E-05±1.69E-04 | 2.31E-03±2.13E-03** | 3.21E-03±3.74E-03 |
| *D_0__Bacteria; D_1__Firmicutes; D_2__Clostridia; D_3__Clostridiales; D_4__Ruminococcaceae; D_5__Ruminiclostridium 6* | 2.08E-03±1.44E-03 | 0** | 0 |
| *D_0__Bacteria; D_1__Firmicutes; D_2__Clostridia; D_3__Clostridiales; D_4__Ruminococcaceae; D_5__Ruminococcaceae UCG-013* | 1.45E-03±1.67E-03 | 0** | 6.79E-05±1.12E-04 |
| *D_0__Bacteria; D_1__Firmicutes; D_2__Clostridia; D_3__Clostridiales; D_4__Ruminococcaceae; D_5__Ruminococcus 2* | 9.18E-03±9.63E-03 | 1.04E-05±3.30E-05** | 5.16E-05±8.72E-05 |
| *D_0__Bacteria; D_1__Firmicutes; D_2__Erysipelotrichia; D_3__Erysipelotrichales; D_4__Erysipelotrichaceae; D_5__Allobaculum* | 1.51E-03±7.18E-04 | 2.39E-01±1.71E-01** | 1.18E-01±9.98E-02 |
| *D_0__Bacteria; D_1__Firmicutes; D_2__Erysipelotrichia; D_3__Erysipelotrichales; D_4__Erysipelotrichaceae; D_5__Dubosiella* | 1.21E-04±1.06E-04 | 4.45E-03±5.45E-03** | 2.10E-02±4.89E-02 |
| *D_0__Bacteria; D_1__Firmicutes; D_2__Erysipelotrichia; D_3__Erysipelotrichales; D_4__Erysipelotrichaceae; D_5__Faecalitalea* | 5.06E-05±8.18E-05 | 3.04E-03±4.05E-03** | 5.78E-03±9.59E-03 |
| *D_0__Bacteria; D_1__Firmicutes; D_2__Erysipelotrichia; D_3__Erysipelotrichales; D_4__Erysipelotrichaceae; D_5__[Clostridium] innocuum group* | 0 | 6.05E-04±4.32E-04** | 8.83E-04±1.07E-03 |
| *D_0__Bacteria; D_1__Proteobacteria; D_2__Deltaproteobacteria; D_3__Desulfovibrionales; D_4__Desulfovibrionaceae; D_5__Desulfovibrio* | 1.54E-02±1.55E-02 | 9.44E-04±1.61E-03** | 1.24E-03±1.02E-03 |
| *D_0__Bacteria; D_1__Tenericutes; D_2__Mollicutes; D_3__Mollicutes RF39; __; __* | 2.03E-02±2.10E-02 | 2.12E-05±4.46E-05** | 1.36E-05±4.29E-05 |

Table S3. RSD values for the identified metabolites in QC samples

| No. | Metabolite identification | RSD of retention times (%) | RSD of peak intensities (%) |
| --- | --- | --- | --- |
| 1 | Alanine | 0.5525 | 21.02 |
| 2 | Glutamine | 0.4662 | 12.86 |
| 3 | L-carnitine | 1.3605 | 8.50 |
| 4 | tyrosine | 1.2974 | 4.57 |
| 5 | Butyrylcarnitine | 0.6704 | 8.24 |
| 6 | Tryptophan | 0.4742 | 13.4 |
| 7 | Indoleacrylic acid | 0.6388 | 9.84 |
| 8 | Valerylcarnitine | 0.1000 | 14.40 |
| 9 | 11-Dehydrothromboxane B2 | 0.082 | 19.67 |
| 10 | LPA (8:0/0:0) | 0.0682 | 22.43 |
| 11 | 3-Hydroxysebacic acid | 0.0652 | 28.41 |
| 12 | Sulfolithocholylglycine | 0.1019 | 20.33 |
| 13 | Taurochenodeoxycholic acid | 0.1118 | 24.33 |
| 14 | LPC (15:0) | 0.1011 | 21.50 |
| 15 | Glycocholic acid | 0.0625 | 20.94 |
| 16 | 20-hydroxy-leukotriene B4 | 0.0884 | 17.12 |
| 17 | 3-oxocholic acid | 0.0606 | 17.79 |
| 18 | Prostaglandin I2 | 0.0606 | 9.34 |
| 19 | 3-oxo-4,6-choladienoic acid | 2.9861 | 19.53 |
| 20 | 7alpha-hydroxy-3-oxochol-4-en-24-oic Acid | 0.0606 | 13.57 |
| 21 | Leukotriene E3 | 0.0515 | 26.71 |
| 22 | 3-xix-7-hydrixychol-4-enoic acid | 0.2229 | 20.73 |
| 23 | Leukotriene B4 | 0.0576 | 20.35 |
| 24 | PC (18:0/22:6) | 0.6229 | 21.73 |
| 25 | Glycoursodeoxycholic acid | 0.0552 | 21.66 |
| 26 | Tetradecanoylcarnitine | 1.1259 | 17.69 |
| 27 | Sphingosine | 1.0164 | 24.10 |
| 28 | Oleamide | 0.2008 | 37.26 |
| 29 | 13-HDoHE | 0.0521 | 14.53 |
| 30 | LysoPE (16:0/0:0) | 0.0862 | 15.41 |
| 31 | 18-hydroxycortisol | 0.2951 | 26.54 |
| 32 | LysoPC (16:1) | 0.0836 | 10.74 |
| 33 | LysoPE (18:1/0:0) | 0.0504 | 36.26 |
| 34 | Palmitoylcarnitine | 1.3286 | 29.38 |
| 35 | Stearoylcarnitine | 1.4858 | 29.10 |
| 36 | Eicosapentaenoic acid | 0.1024 | 15.48 |
| 37 | 5-HPETE | 0.1023 | 22.74 |
| 38 | DG(14:0/18:1/0:0) | 0.0759 | 20.23 |
| 39 | LysoPC (16:0) | 0.0984 | 27.49 |
| 40 | LysoPC (20:2) | 0.0981 | 29.84 |
| 41 | 15S-HETrE | 0.0743 | 72.41 |
| 42 | Linolenic acid | 0.0689 | 37.15 |
| 43 | Docosahexaenoic acid | 0.1207 | 24.64 |
| 44 | Arachidonic acid | 0.1211 | 42.17 |
| 45 | Stearic acid | 0.1276 | 8.75 |
